# Pathogenesis and Research Models of Acute Influenza-Associated Encephalitis/Encephalopathy: An Update

**DOI:** 10.3390/v18010095

**Published:** 2026-01-09

**Authors:** Jintian Wei, Haoying Huang, Xiaohuan Wu, Yi Xu, Xiaohui Wang

**Affiliations:** 1 State Key Laboratory of Respiratory Disease, Guangzhou Institute of Pediatrics, Guangzhou Women and Children’s Medical Center, Guangdong Basic Research Center of Excellence for Respiratory Medicine, Guangzhou Medical University, Guangzhou 510623, China; 2023210611@stu.gzhmu.edu.cn (J.W.); haoying_huang@163.com (H.H.); 2 Translational Medical Center for Stem Cell Therapy, Institute for Regenerative Medicine, Shanghai East Hospital, School of Life Sciences and Technology, Tongji University, Shanghai 200127, China; 3 Shanghai Key Laboratory of Signaling and Disease Research, Frontier Science Center for Stem Cell Research, School of Life Sciences and Technology, Tongji University, Shanghai 200092, China; 4 Department of Infectious Disease, Guangzhou Women and Children’s Medical Center, Guangzhou Medical University, Guangzhou 510120, China; xiaohuanwu2022@163.com (X.W.); xuyi70@163.com (Y.X.)

**Keywords:** influenza virus, influenza associated encephalitis/encephalopathy, neurological lesion, research model

## Abstract

Influenza-associated encephalitis/encephalopathy (IAE) is a severe neurological complication characterized by central nervous system dysfunction and structural damage following influenza virus infection. Predominantly affecting infants and young children, IAE exhibits its highest incidence in those under five years of age. Key clinical manifestations of IAE include acute seizures, sudden high fever, and impaired consciousness, frequently progressing to coma. Neuroimaging, particularly magnetic resonance imaging (MRI), often reveals multifocal brain lesions involving multiple brain regions, including the cerebellum, brainstem, and corpus callosum. The prognosis of IAE is poor, with a mortality rate reaching 30%. Current diagnosis relies heavily on clinical presentation and characteristic neuroimaging findings, as the precise pathogenesis of IAE remains elusive. While various research models, including cell lines, brain organoids, and animal models, have been developed to recapitulate IAE features, significant limitations persist in modeling the core clinical pathophysiology observed in pediatric patients, necessitating further model refinement. This review synthesizes the clinical spectrum of IAE, summarizes progress in understanding its pathogenesis, and critically evaluates existing research models. We aim to provide a foundation for utilizing experimental approaches to elucidate IAE mechanisms and identify potential therapeutic strategies.

## 1. Introduction

Influenza viruses are single-stranded negative-sense RNA viruses belonging to the Orthomyxoviridae family, primarily comprising influenza A virus (IAV), influenza B virus (IBV), influenza C virus (ICV), and influenza D virus (IDV). IAV, IBV, and ICV can all cause human infection, whereas IDV has so far only been reported to infect pigs and cattle [1]. As highly contagious respiratory pathogens, IAV and IBV primarily caused human influenza epidemics, typically occurring annually in winter and spring. Predominant circulating strains include influenza A subtypes (H1N1 and H3N2) and influenza B lineages (B/Victoria and B/Yamagata) [2]. According to World Health Organization (WHO), seasonal influenza viruses infect approximately one billion people worldwide each year, resulting in over three million severe cases and contributing to up to 650,000 respiratory-related deaths [3]. Therefore, it represents a significant global public health threat.

Although influenza viruses infect the respiratory tract system, they can also cause complications in other extrapulmonary organs, including the heart, brain, intestines, stomach, and kidneys [4,5,6]. Among the most severe complications is influenza-associated encephalitis/encephalopathy (IAE). IAE primarily affects children, with the highest incidence (64.8%) in those under five years old, and is caused by both IAV and IBV. The high incidence of infectious encephalitis and encephalopathy in children is attributed to their still-developing physiological structure. Upon initial exposure to potent pathogens such as influenza, children’s nascent immune systems are prone to dysregulated activation, which can trigger a systemic cytokine storm. Furthermore, the underdeveloped blood–brain barrier (BBB) and vulnerable brain tissue are more easily compromised by elevated levels of inflammatory factors and viral invasion, ultimately promoting neuroinflammation and brain injury [7]. IAV demonstrates greater pathogenicity, leading to a higher proportion of pediatric IAE cases compared to IBV. The peak occurrence of IAE aligns with the influenza epidemic seasons during winter and spring [5,8]. Reported incidence and mortality rates for IAE vary across different countries and regions. A study conducted by a Class A tertiary hospital in China found that among 446 children with laboratory-confirmed influenza virus infection, 71 cases were diagnosed with IAE, representing an incidence rate of 15.9%. With twelve fatalities, the mortality rate was 16.9% [5]. According to a pediatric hospital in Italy, 13.1% of children with influenza infection exhibited symptoms of IAE, with no associated deaths [9]. A nationwide survey in Japan found that the seasonal incidence rate of IAE in the general population was 2.3 cases per million people. The incidence was highest among children aged 2–4 years (3.8–6.0 cases per million), while the rate among adults was considerably lower (0.19 cases per million) [10]. An epidemiological study indicates that in Australia, the average annual incidence rate of IAE among children (aged 14 years or younger) stands at 2.8 cases per 100,000, whereas in the United States, the average incidence rate is 4 cases per 100,000 [11,12]. The prognosis for IAE remains poor, and specific treatments are currently unavailable. Common management strategies focus on symptom control and include antiviral medications (oseltamivir/peramivir), often combined with high-dose methylprednisolone, intravenous immunoglobulin (IVIG), and plasma exchange [13,14,15].

This review aims to systematically generalize about the clinical syndromes and recent advances in IAE pathogenesis research, and evaluate relevant experimental models, including in vitro cell lines, organoids and animal models. We highlight the connections between the clinical manifestations of IAE disease and its underlying pathogenic mechanisms, which provide a valuable reference for subsequent investigations into its pathogenesis and clinical therapeutic strategies.

## 2. Pathological Characteristics and Diagnosis of IAE

IAE is a rapidly progressive neurological complication occurring in pediatric patients following influenza infection, typically manifesting within several days of initial influenza symptoms. Its hallmark presentation includes persistent seizures accompanied by diminished consciousness, ranging from somnolence to coma [5,16,17,18,19]. Diagnostic neuroimaging with magnetic resonance imaging (MRI) often reveals hyperintense signals in the brainstem, thalamus, cerebellum, periventricular white matter, and cortex, indicative of cerebral edema or swelling. Electroencephalograms (EEGs) may appear normal or demonstrate slow-wave activity [5]. Laboratory findings include elevated procalcitonin and C-reactive protein levels in some patients, while cerebrospinal fluid protein levels can be either elevated or within normal limits [20]. Based on MRI characteristics and clinical symptoms, clinicians classify IAE into several distinct clinical syndromes: acute necrotizing encephalopathy (ANE), acute encephalopathy with biphasic seizures and late diffusion restriction (AESD), mild encephalitis/encephalopathy of a reversible splenial lesion (MERS), hemorrhagic shock and encephalopathy syndrome (HSES), posterior reversible encephalopathy syndrome (PRES) and acute disseminated encephalomyelitis (ADEM) (Table 1) [21].

ANE is the most severe clinical syndrome of IAE. Its defining characteristics include rapid deterioration of clinical condition, parenchymal necrotic lesion, high mortality rates, and poor prognosis [22], making it a leading cause of influenza-related death in children [23]. Characteristic MRI findings reveal bilateral thalamus, brainstem, cerebellum, and periventricular white matter lesions in the pediatric patients [24,25,26]. Pathological studies indicate that ANE involves vascular endothelial lesions, localized hemorrhage and brain tissue necrosis, accompanied by cerebral edema [27].

AESD is a prevalent IAE subtype, particularly in Japanese children [28]. Initial symptoms typically include a persistent fever and convulsions in affected infants, rapidly progressing to include epilepsy and impaired consciousness. Brain MRI usually appears normal within the first two days post-infection; however, reduced subcortical diffusion and a “bright tree appearance” become evident between days 4 and 6, ultimately progressing to cerebral atrophy [28,29,30,31]. Despite a relatively lower mortality rate compared to ANE, AESD carries a poor prognosis, frequently resulting in persistent neurological sequelae of varying severity [32,33].

MERS primarily presents with epileptic seizures, sometimes accompanied by fever. Brain MRI reveals a distinctive high-signal-intensity in the corpus callosum, with lesions predominantly concentrated in the callosal body region [34,35]. Cerebrospinal fluid analysis shows normal cellular and protein levels. Following treatment, affected children usually recover within two months, experiencing a favorable prognosis and no residual neurological sequelae [36,37,38].

HSES represents another major acute encephalopathy subtype associated with influenza. Patients may experience symptoms like impaired consciousness, convulsions, and shock within one day of infection, followed by rapid progression accompanied by diarrhea, hepatic and renal dysfunction, disseminated intravascular coagulation (DIC), and acute cerebral edema [39,40]. This disorder has a high mortality rate and a poor prognosis, with affected patients often experiencing residual neurological sequelae following treatment [41,42].

The clinical manifestations of PRES include headache, seizures, visual disturbances, and focal neurological deficits. The majority of patients experience elevated arterial blood pressure, and some of them develop hypertensive emergencies. Typical MRI findings demonstrate bilateral symmetrical vascular edema affecting both cortical and subcortical deep white matter, with lesions commonly involving the occipital and parietal lobes [43,44,45]. This condition has a good prognosis, with neuroimaging changes being reversible [46].

ADEM is an encephalitic disorder associated with spinal cord demyelination. Neurological symptoms, such as headache, vomiting, pseudomeningitis, behavioral changes, decreased alertness, multifocal neurological deficits, and encephalitic features like irritability, confusion, somnolence, and coma, usually appear in pediatric patients within one to two weeks of infection. Neuroimaging and laboratory tests are the mainstays of diagnosis. Brain MRI reveals multiple ill-defined or asymmetrical white matter lesions, though without involvement of the periventricular white matter regions; spinal MRI shows abnormal hyperintensity in the central grey matter and peripheral white matter of some segments. Elevated protein and cell counts in cerebrospinal fluid are frequently found in laboratory studies. Serum testing for myelin-associated glycoprotein (MOG) and neuromyelitis optical (NMO) and antibodies enhances the diagnostic sensitivity and specificity for central nervous system (CNS) demyelinating disorders [47,48,49,50]. ADEM usually does not require long-term treatment, with a generally favorable overall prognosis. Between 23% and 100% of affected children achieve complete recovery, though approximately 27% experience recurrent or biphasic ADEM [51].

## 3. Pathophysiology and Disease Mechanisms

**Figure 1:**
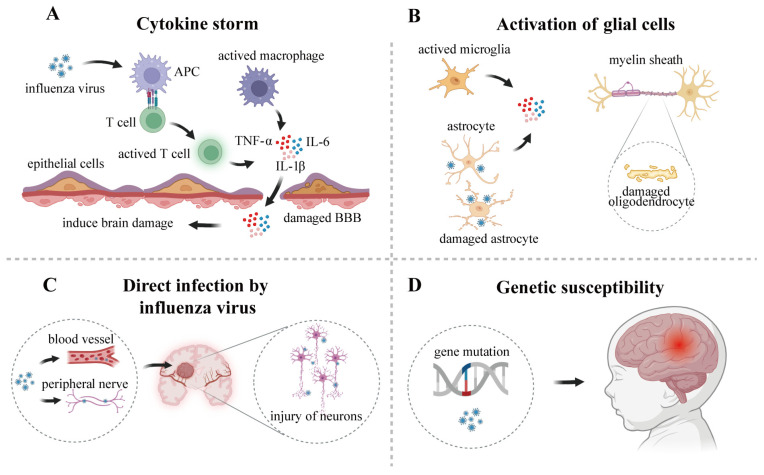
A summary of the potential pathogenesis of IAE. (**A**). Cytokine storm: Influenza virus infection triggers immune cell activation and systemic cytokine storms, characterized by high levels of inflammatory mediators such as TNF-α, IL-6, and IL-1β infiltrating brain tissue, which alters BBB permeability and induces the development of encephalitis. (**B**). Activation of glia cells: Influenza virus infection induces activation of microglia and astrocytes, leading to the secretion of pro-inflammatory cytokines. Infected astrocytes also exhibit podocyte breakage and cellular vacuolization. Influenza virus infection also disrupts the homeostasis of oligodendrocytes, affecting myelin synthesis and resulting in cognitive and behavioral alterations. (**C**). Direct infection by influenza virus: Influenza viruses can enter brain tissue via the bloodstream or by infecting peripheral nerves, directly infecting neurons and promoting brain tissue damage. (**D**). Genetic susceptibility: Mutations in certain susceptibility genes may predispose pediatric influenza patients to the occurrence of IAE.

After outlining the clinical spectrum of IAE—from its general manifestations to the distinct features and prognosis of its major subtypes (ANE, AESD, MERS, HSES, PRES, and ADEM)—a central question emerges: what are the underlying pathological mechanisms that induce the development of IAE? Although the exact mechanisms remain incompletely elucidated, current evidence points to several interconnected pathways. These include immune dysregulation (notably cytokine storm), activation of glial cells in the CNS, potential routes for direct viral invasion into brain tissue, and individual genetic susceptibility (Figure 1).

### 3.1. Cytokine Storm

Cytokine storm is one of the most important pathogenic mechanisms of IAE. Following infection of the respiratory mucosa, influenza viruses induce the production of multiple pro-inflammatory cytokines, including tumor necrosis factor (TNF), interferon (IFN), and interleukin (IL). Under normal physiological conditions, these cytokines assist in eliminating invading pathogens by regulating immune responses. However, when the body’s immune defense mechanisms become overactivated, this leads to excessive cytokine release, thereby triggering cytokine storm [23].

Although cytokine storm enhances pathogen clearance, it also induces severe systemic inflammatory responses, which can lead to multi-organ dysfunction and even life-threatening conditions. Previous clinical data have shown that serum and cerebrospinal fluid levels of cytokines were significantly elevated in IAE patients, with particularly high levels of IL-6, IL-1β, and TNF-α [52,53]. These cytokines may also promote neuronal damage by disrupting the integrity of the BBB, thereby facilitating the infiltration of inflammatory mediators and immune cells into the CNS. Specifically, cytokines can compromise the barrier function of the BBB by downregulating the expression of tight junction proteins such as Occludin, Claudin-5, and Zonula occludens-1 (ZO-1), which allows cytokines like IL-6 and TNF-α, along with activated immune cells, to enter brain tissue from the bloodstream (Figure 1A) [54,55]. These studies further indicate that cytokine storm play a significant role in the development and progression of IAE.

### 3.2. Activation of Glial Cells

Microglia, astrocytes, and oligodendrocytes are the three main types of glial cells within the CNS, each with distinct roles in CNS development and pathological progression [56]. As the primary responders to neuroinflammation or injury, microglia can rapidly sense and adapt to changes in the brain microenvironment. As resident macrophages in the CNS, microglia exhibit rapid responsiveness to pathogens invading the brain parenchyma, including viruses, bacteria, fungi, and parasites, contributing to immune defense by releasing pro-inflammatory cytokines [57,58,59]. Additionally, microglia can interact with CD8^+^ and CD4^+^ T cells to eradicate infections through antigen presentation via major histocompatibility complex (MHC) class I and II [60].

Astrocytes are among the most abundant glial cell types in the CNS, with functions encompassing ion concentration regulation, neurotransmitter and neurohormone secretion, maintenance of BBB formation and integrity, and participation in neurogenesis. Astrocytes respond to various CNS injuries and diseases by changing morphologically and functionally to become reactive astrocytes [61,62].

Oligodendrocytes are key components in the myelin sheath formation of neuronal axons, which is essential for efficient electrical signal transduction in the CNS. Furthermore, oligodendrocytes provide metabolic support to neurons by producing multiple neurotrophic factors. Notably, oligodendrocytes secrete immunomodulatory molecules such as complement factors, chemokines, and inflammatory cytokines, thereby participating in neuroinflammatory responses [63,64].

Studies have shown that IAV can induce microglia proliferate and activation. Subsequently, inflammatory mediators such as TNF-α, IL-6, IL-1, and complement component 3 (C3) are produced, causing excessive neuronal excitation and damage to adjacent neurons. This process is considered a key factor triggering viral encephalitis [58,59,65]. Astrocytes can also be infected by IAV, leading to cytopathic changes such as cell rounding and vacuolization (Figure 1B) [66]. A post-mortem report on an IAE case revealed proliferative reactive astrogliosis in the patients, with disruption observed in the terminal processes around blood vessels [67]. Furthermore, infected astrocytes upregulate pro-inflammatory cytokine expression, triggering inflammatory cascades that contribute to neurological complications [68,69].

Influenza virus infection damages oligodendrocytes, downregulates transcripts associated with myelin formation, alters the myelin lipidome, and impairs myelinogenesis, potentially leading to cognitive and behavioral abnormalities [70,71,72,73]. However, the mechanism by which glial cells, particularly immune-competent activated microglia, induce IAE remains unclear.

### 3.3. Direct Infection by Influenza Virus

The question of whether the influenza virus directly enters the CNS to cause brain damage has been a key focus of research and discussion regarding the pathology of IAE. Two independent studies conducted in 2012 and 2021 performed quantitative PCR analyses on cerebrospinal fluid (CSF) samples from IAE patients. Neither study detected viral nucleic acids, suggesting that direct CNS infection by influenza viruses may not be the primary pathogenic factor in the cases examined [20,44]. However, Santini et al. reported three cases of influenza encephalitis in 2009 where viral RNA was detected in CSF [74]. This finding supports the possibility that direct viral invasion of brain tissue represents another potential pathogenesis mechanism for IAE, suggesting that influenza viruses can directly enter the CNS in some cases [75,76,77].

Current understanding identifies the primary pathways for CNS invasion by influenza virus include: (1) the hematogenous route, where virions cross the BBB by the circulatory system and penetrate the brain parenchyma; (2) the neural pathway-mediated route, involving anterograde or retrograde axonal transport through peripheral nerves such as the vagus, trigeminal, olfactory and nerves (Figure 1C) [78,79].

The influenza virus can facilitate hematogenous invasion by compromising BBB integrity. Similar to the mechanism by which cytokine storm affects the BBB, the influenza virus itself can downregulate the key tight junction proteins on cerebral microvascular endothelial cells, thereby increasing BBB permeability. In vitro studies by Lei et al. showed that H7N9 infection of human brain microvascular endothelial cells significantly downregulated the expression of tight junction proteins Claudin-5, Occludin, and vascular endothelium-cadherin, thereby disrupting BBB integrity and increasing permeability [80].

In addition to the hematogenous route, the influenza virus may also invade the CNS via neural pathways, primarily through anterograde or retrograde axonal transport along cranial nerves. Multiple in vivo animal studies confirm the olfactory pathway as an important route for peripheral influenza virus to invade the CNS. For instance, SHINYA et al. employed a ferret model for intranasal highly pathogenic H5N1 infection and utilized 3D imaging techniques to trace neural pathways from the olfactory bulb to the cortex. Their study revealed the viral antigen distribution and brain lesions along the olfactory pathway, indicating that viral entry into the brain parenchyma via this route can induce non-purulent inflammation [81]. Highly pathogenic avian influenza viruses (HPAIV) evolve from low pathogenic precursors via mutations, primarily through the acquisition of a multibasic cleavage site (MBCS) in the haemagglutinin (HA) glycoprotein. This structural alteration enables cleavage by ubiquitously expressed furin-like protease in the host, thereby enhancing viral virulence and promoting systemic dissemination [82,83]. Experiments by Schrauwen et al. demonstrated that HPAI H5N1 strains lacking MBCS were restricted to respiratory tract infection and failed to spread systemically, including to brain tissue, underscoring the critical role of this cleavage site in neuroinvasiveness [84]. Furthermore, animal studies indicate that the influenza virus can invade the CNS by infecting peripheral nerves such as the trigeminal and vagus nerves. The virus initially replicates within respiratory tract epithelial cells such as in the nasopharynx, trachea, bronchi, or alveoli, before invading adjacent ganglia such as the vagal ganglion. Virions can then ascend along axons, traversing structures like the solitary tract nucleus to reach the brainstem and higher centers, ultimately causing intracranial infection [78,85,86].

Invasion of the CNS by influenza virus involves multiple pathways, including hematogenous and neural routes. These potential mechanisms can act synergistically to contribute to the pathogenesis of influenza-associated encephalopathy. Further clinical diagnostic data and animal model studies are required to elucidate the pathogenic mechanisms.

### 3.4. Genetic Susceptibility

Genetic predisposition also contributes to the pathogenesis of IAE, exhibiting a hereditary component. Research indicates that mutations in the carnitine palmitoyltransferase II (*CPT II*) gene represent a risk factor for IAE. Shinohara et al. discovered that the *CPT II* p.F352C mutation was significantly more prevalent in patients with AESD and ANE compared to healthy individuals. This finding suggests the mutation plays a key role in the pathogenesis of acute encephalopathy (Figure 1D) [87].

*CPT II* is a critical mitochondrial enzyme regulating fatty acid oxidation and essential for maintaining mitochondrial energy homeostasis. *CPT II* mutations cause the enzyme protein to exhibit thermal instability, reduced basal activity, and shortened half-lives. Under hyperthermic conditions, patients carrying this mutation experience a significant further decline in *CPT II* enzyme activity. This impairs mitochondrial fatty acid oxidation for energy production, creating an energy deficit that may induce hypoxic damage to the BBB, leading to cerebral edema and ultimately acute encephalopathy [88].

Further research by Shinohara et al. indicates that specific homozygous genotypes of the adenosine A2A receptor gene (*ADORA2A*) also constitute genetic risk factors for AESD, with a pathogenic mechanism linked to neuroexcitotoxicity. Through analysis of *ADORA2A* single-nucleotide polymorphisms (SNPs) in 85 AESD patients, they found individuals carrying the homozygous ADORA2A genotype exhibited a significantly elevated risk of development of AESD [89]. Subsequent investigations revealed that the overexpression of *ADORA2A* protein in brain tissue and lymphoblastoid cell lines activates adenosine/cAMP signaling cascades, promoting the release of excitatory neurotransmitters. This process ultimately triggers epileptic seizures and exacerbates excitotoxic neuronal damage, contributing to the pathogenesis of AESD [89,90].

Another study identified mutations in the serine/threonine kinase 39 gene (*STK39*), located on chromosome 2q24.3, as associated with the AESD occurrence, presenting a new susceptibility gene. The mutated *STK39* protein activates the p38 mitogen-activated protein kinase (MAPK) pathway, which influences inflammatory response regulation and may contribute to AESD development [91].

In 2003, Neilson et al. discovered that ANE exhibits familial inheritance, identifying an autosomal dominant form [92]. Subsequent clinical cases revealed that familial ANE is caused by mutations in the gene RAN binding protein 2 (*RANBP2*). Patients exhibit missense mutations leading to erroneous amino acid coding. For example, the most common genetic mutation site is c.1754 C > T, where threonine at position 585 is replaced by methionine (p.Thr 585 Met) [93,94,95]. Currently, several new mutation sites have been identified, such as c.7454 G > T, c.7474 A > G, c.7807 C > T, c.7918 C > A, c.9041 A > G, and c.872 A > G [95,96]. This *RANBP2*-associated ANE is termed ANE1 [97].

*RANBP2* is a nuclear pore complex protein located at the nuclear pore surface, participating in the modification of proteins entering and exiting the nuclear pore. It plays a role in nucleocytoplasmic transport and glycolysis regulation. Mutations disrupt these functions, leading to energy metabolism dysregulation during acute infection [98]. In vitro findings indicate the RanBP2 protein inhibits IL-6 mRNA translation, suppressing IL-6 protein expression levels. Conversely, mutations of RanBP2 relieve this inhibition, upregulating IL-6 protein expression. These studies suggest RanBP2 mutations may dysregulate cytokine expression, promoting excessive inflammatory responses and contributing to ANE1 progression [99].

Different subtypes of IAE involve distinct pathogenic mechanisms. ANE arises primarily from a systemic inflammatory cytokine storm triggered by influenza infection, coupled with genetic susceptibility. A specific form, ANE1, is directly linked to mutations in *RANBP2*, which caused dysregulated expression of inflammatory cytokines such as IL-6 [98,99,100,101]. Clinically, this manifests as severe systemic inflammation and symmetric necrotic lesions, illustrating how genetic predisposition and cytokine storm interact. AESD may result from genetic mutations that increase neuronal vulnerability during febrile infection: for example, *CPT II* mutations disrupt energy metabolism, while *ADORA2A* variants enhance excitotoxicity [29,87,89,102]. These mechanisms explain the characteristic biphasic course—initial seizures due to excitotoxicity, followed by delayed neurological impairment associated with cytotoxic edema and energy failure. MERS, a milder encephalitis variant, is thought to involve transient, localized cytokine-mediated blood–brain barrier dysfunction and glial activation, reflecting a reversible inflammatory process without necrosis [103]. HSES is driven by systemic shock, disseminated intravascular coagulation (DIC), and multiorgan failure resulting from an overwhelming cytokine storm [104,105,106]. ADEM, in contrast, resembles a virally triggered autoimmune demyelinating disorder of the CNS [48,107].

The pathogenesis of IAE is multifactorial, often involving synergistic interactions among several elements. Specific gene mutations, such as those in *RANBP2*, *CPT II*, *ADORA2A*, and *STK39*, constitute the “first hit”, lowering the disease threshold through various mechanisms: *RANBP2* mutations disrupt inflammatory control; *CPT II* mutation defects predispose neurons to energy failure during fever, and *ADORA2A* mutations enhance excitotoxic susceptibility [29,87,89,98,99,100,101,102]. Influenza infection then acts as a “second hit”, inducing a systemic cytokine storm that disrupts the BBB and facilitates the entry of immune cells and inflammatory mediators into brain tissue [52,53,54,55]. Viral invasion may occur via hematogenous dissemination following systemic infection or through neural routes such as the olfactory or trigeminal nerves. Once inside the CNS, the virus can activate glial cells, further amplifying neuroinflammation [58,59,65,66,67,68,69,74,75,76,77]. The convergence of genetic vulnerability and infection-mediated inflammation thus synergistically drives the development and severity of encephalopathy.

## 4. Research Progress on IAE-Related Models

**Figure 2:**
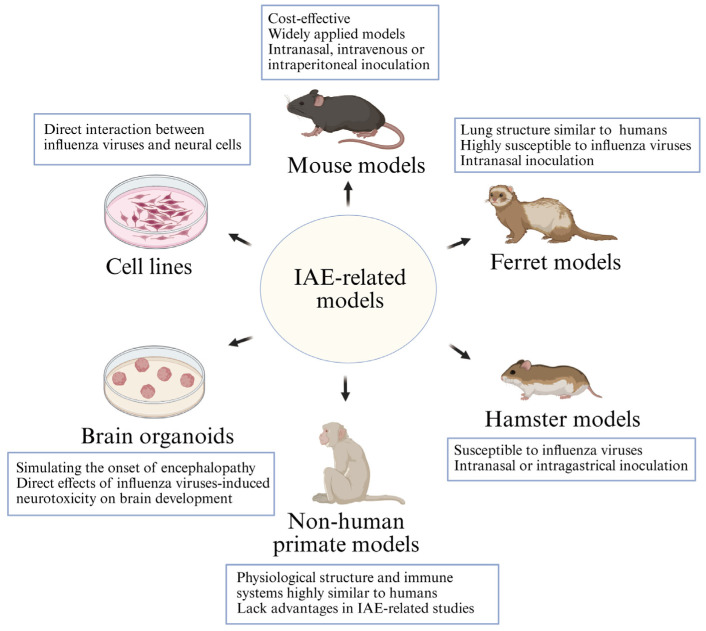
Characteristics concerning IAE-related in vitro cell lines, brain organoids, and animal models. Cell lines are frequently used to investigate the direct interactions between influenza viruses and various neural cells, including neurons and glial cells. Brain organoids can more effectively simulate influenza infection of brain tissue in vitro, thereby inducing the occurrence of brain damage. Animal models include mouse models, ferret models, hamster models, and non-human primate models. Mouse models are commonly used as animal models for investigating the pathogenesis of IAE, being cost-effective and widely utilized. Common methods for inducing IAE infection include intranasal inoculation, intravenous inoculation, and intraperitoneal infection. Ferrets are highly susceptible to influenza viruses, and their pulmonary physiology closely resembles that of humans, making them an excellent animal model for investigating influenza infection and the development of encephalopathy. Hamsters are also animals susceptible to influenza viruses, and it has been discovered that intranasal and intragastric infection can cause neurological alterations in hamsters. Non-human primate models exhibit high similarity to humans in physiological structure and immune systems, yet they lack advantages in investigating encephalitis/encephalopathy caused by influenza viruses.

The pathogenesis of IAE is thought to involve multiple factors such as cytokine storms, glial cell activation, potential viral neural invasion, and genetic susceptibility, forming a complex framework of interactions. However, elucidating the interactions of these pathogenic factors requires establishing reliable experimental systems that can faithfully recapitulate the human disease. The absence of such models remains a major obstacle to investigating the onset and progression of IAE. The following section will systematically review current experimental approaches, including cell lines, human brain organoids, and animal models, which serve as essential tools for validating pathogenic pathways and exploring the pathophysiology of IAE (Figure 2 and Table 2).

### 4.1. In Vitro Cell Line Models

Cellular-level research on IAE primarily focuses on the direct interaction between influenza viruses and neural cells, particularly viral infectivity towards neural cells. Several in vitro experiments have demonstrated that various influenza viruses (including H1N1and H7N7) can infect microglia and replicate within these cells. This process promotes the upregulation of pro-inflammatory cytokines in microglia, thereby inducing inflammatory responses [108,109]. Different viral strains exhibit varying capacities to infect neurons and glial cells. Wu et al. found that, although the avian influenza H5N1 virus can simultaneously infect human astrocytes and neuronal cells, its replication capacity is confined to astrocytes, failing to produce progeny viruses within neuronal cells [110]. In contrast, the avian influenza H7N9 virus exhibits opposite infectivity characteristics: it can effectively replicate and propagate within human neuronal cells yet fails to produce progeny viruses within infected human astrocytes [68].

Furthermore, Wang et al. infected primary cortical neuronal cells of mice with the virus strains A/Puerto Rico/8/1934 (H1N1) and A/Shantou/169/2006 (H1N1). Results showed that both viruses exhibited limited infectivity towards cortical neurons, failing to replicate efficiently and exerting minimal impact on cellular viability but significantly upregulated the expression levels of inflammatory cytokines in these cells [111]. Collectively, these findings demonstrate that influenza virus infection of neural cells induces elevated expression of inflammatory cytokines, promotes inflammatory responses, and leads to cytopathic effects or apoptosis. However, they also reveal significant differences in the infectivity of distinct influenza virus subtypes toward neural cells. This provides a crucial basis for selecting viral subtypes in subsequent animal models and further exploration of disease mechanisms.

### 4.2. Brain Organoid Models

With rapid advancements in stem cell biology, organoids derived from human pluripotent stem cells (hPSCs) have emerged as invaluable tools for modeling human diseases. Human brain organoid models, which comprise progenitor cells, neurons, and glial cells, can self-assemble into three-dimensional (3D) aggregates with well-organized structures [112]. Zhang et al. used human brain organoid models to investigate the direct impact by influenza virus-induced neurotoxicity on brain development. They discovered the influenza virus strain A/WSN/1933 (H1N1) could infect multiple neural cell types, including SOX2^+^ neural stem cells (NSCs), MAP2^+^ neurons, and GFAP^+^ astrocytes. Concurrently, the virus upregulated the expression of inflammatory cytokines, such as IL-6, TNF-α, and IFN-γ, thereby inducing apoptosis in NSCs and neuronal cells. This ultimately resulted in impaired development of the brain organoids [113].

### 4.3. IAE-like Animal Models

Animal models are indispensable research tools for simulating human disease progression and investigating pathogenic mechanisms. This section reviews the primary animal models of IAV-induced neurological disorders, including mice, ferrets, and other relevant models, focusing on the establishment methods of different models and the pathological features they induce.

#### 4.3.1. Mouse Models

Experimental mice, owing to their low cost and high cost-effectiveness, have become one of the most widely used influenza infection models for influenza infection and a common species for investigating the pathogenesis of IAE. In mouse models, IAE-like symptoms are typically induced through three primary routes of administration: intranasal inoculation, intravenous inoculation, and intraperitoneal infection. Among these, C57BL/6 and BALB/c are the most commonly employed mouse strains. It is noteworthy that the pathology is influenced by multiple factors, including viral subtypes, inoculum dose, and the age/development stage of the mice.

Intranasal infection is the most frequently used method for inducing IAE-like mouse models and is regarded as a means to model complications arising from induced respiratory symptoms [114,115,116,117]. As previously stated, the HA surface of HPAIV contains MBCS, enabling it to be readily cleaved by enzymes within the host organism, thereby acquiring the capacity for systemic infection [82]. In investigating the pathogenic characteristics of the HPAI H7N9 virus, Wu et al. observed that 6–8 week-old BALB/c mice, following intranasal infection with H7N9, developed extrapulmonary organ infections, including in the brain and heart. Further investigations revealed inflammatory cell infiltration in the brain on days 2, 4, and 6 post-infection, manifesting as moderate encephalitis, with substantial viral antigens detectable in brain tissue by day 6 [115,117]. However, influenza strains of distinct subtypes exhibit marked differences in their ability to induce encephalitis. Gu et al. infected BALB/c mice with A/Puerto Rico/8/1934 (H1N1) and observed that during the acute phase of viral infection (3 and 7 days post-inoculation, dpi), no significant upregulation of inflammatory factors was detected in the brain. During the recovery phase (14 dpi), however, significant increases in chemotactic cytokines (CCL-2, CCL-3, CCL-5, and CXCL10) and interferon-related molecules (INF-α, IFN-β, and IFN-γ) in the brain were observed, along with high expression of intracerebral cytokines (ionized calcium-binding adapter molecule 1, IBA-1 and brain-derived neurotrophic factor, BDNF) (14 dpi) [118]. Additionally, the intranasal infection mouse model has been employed to investigate post-infection behavioral alterations, including short-term or long-term neuroinflammatory responses induced by non-neurotropic or neurotropic virus strains, morphological changes in hippocampal neurons, and cognitive impairments [65,119,120].

In contrast, intravenous injection of viral particles to induce an IAE mouse model is considered a more efficient approach. Kimura-Ohba et al. successfully induced cerebral edema-like lesions in mice via intravenous injection of A/Puerto Rico/8/1934 (H1N1), with severe neurological symptoms observed within 72 h. This pathological progression closely mirrors the clinical course of acute encephalopathy. Their findings indicate that IAV transmitted via the bloodstream can infect cerebral vascular endothelial cells, triggering necrotic apoptosis and thereby precipitating cerebral edema [67].

Intraperitoneal injection of IAV has been employed to simulate systemic hematogenous dissemination induced by influenza. The A/WSN/1933 (H1N1) strain, through in vitro infection of neuronal cells, induces the conversion of membrane glycoproteins on neuronal surfaces and cellular prion protein (PrP^C^) into scrapie prion protein (PrP^SC^), thereby triggering neurological disorders [121]. Research by Linnéa Asp et al. revealed that intraperitoneal injection of this strain in 4-or 5-day-old newborn mice alters the ornithine pathway, ultimately leading to sensorimotor gating dysfunction in adult mice [122]. Yu et al. employed a peritoneal infection model in 5-day-old BALB/c neonatal mice, finding that viral presence in the brain could be detected as early as 1 dpi. By 5 dpi, viral antigens were detectable in regions including the hippocampus, cerebellum, midbrain, cortex, and medulla. The study further demonstrated that peritoneal infection in neonatal mice impairs myelination and induces alterations in brain function [73,79].

Although laboratory mice offer numerous advantages in influenza virus research, their limitations, such as low susceptibility to natural influenza strains and not being natural hosts for influenza virus, cannot be overlooked.

#### 4.3.2. Ferret Models

Ferrets are a common mammalian model for respiratory infection research. Their small stature and pulmonary physiological structure closely resemble those of humans. Ferrets exhibit high susceptibility to influenza viruses, simulating various post-infection clinical symptoms, such as elevated body temperature, persistent high fever, nasal discharge, and sneezing. Furthermore, infected ferrets can transmit the virus to healthy individuals via airborne routes [123,124,125,126].

Given these characteristics, ferret models are frequently employed to investigate the pathogenesis of IAV. In ferret models, intranasal infection is the most prevalent modeling approach. Studies indicate that different IAV subtypes exhibit varying capacities for CNS infection in ferrets. For instance, Brand et al. observed that HPAIV H5N1, compared to seasonal H3N2 and pandemic H1N1 IAV, exhibits enhanced ability to spread to the CNS [127]. Notably, the H5N1 influenza virus is frequently employed to investigate the invasion of the CNS by influenza viruses and their effects on brain tissue. Multiple studies indicate that intranasally inoculated H5N1 accumulates extensively in the olfactory epithelium, potentially infecting the olfactory bulb via the olfactory system, ultimately threatening brain tissue [81,84,128]. The reason lies in the fact that HPAIV H5N1 possesses the MBCS, enabling it to better invade tissues and organs outside the lung [129].

#### 4.3.3. Hamster Models

Hamsters serve as highly sensitive animal models for influenza viruses. Compared to mice, hamsters exhibit greater susceptibility to human H3N2 influenza viruses and seasonal H1N1 strains, and respiratory infections can be induced without the need for influenza-adapted variants [130,131]. However, studies using hamster models to investigate influenza virus-induced alterations in the CNS remain relatively scarce. Research by SHINYA et al. indicates that the route of transmission and viral strain differences can significantly influence organ infection patterns in hamsters: hamsters intranasally infected with the A/Vietnam/1203/2004 (H5N1) exhibited detectable viral titers in the brain, whereas those infected intragastrically showed no detectable virus. Conversely, both intranasal and intragastric infection with the A/Vietnam/UT3062/2004 (H5N1) resulted in detectable viral titers in the brains of infected hamsters [132]. Although both H5N1 subtypes are considered highly pathogenic strains.

In an experiment comparing the long-term effects of severe acute respiratory syndrome coronavirus 2 (SARS-CoV-2) and IAV on hamsters, it was found that despite undetectable viral RNA in the brains of the IAV group at 3 dpi, IAV infection still significantly impacted related metabolic responses and immune regulation within the brain compared to the SARS-CoV-2 group, affecting multiple brain regions, including the cerebellum, thalamus, striatum, olfactory bulb, and prefrontal cortex, indicating IAV’s potential impact on the CNS [133].

#### 4.3.4. Non-Human Primate Models

Non-human primates (NHP), owing to their genetic characteristics, physiological structure, and immune systems that are highly similar to those of humans, have become crucial tools for investigating the pathogenesis by influenza virus infection. NHPs currently employed in influenza-related research include African green monkeys, rhesus monkeys, macaques, and crab-eating macaques [134]. However, NHP models appear to lack significant advantages in investigating the effects by influenza viruses on the CNS. Studies have found that, in macaques, crab-eating macaques, and marmosets nasally infected with HPAI H5N1, viral antigens are difficult to detect in the brain, despite the virus having spread to other organs beyond the lungs, such as the heart and thoracic lymph nodes [135,136,137]. Although NHPs share substantial physiological traits with humans, their limited manifestation of IAE underscores an essential principle: model utility is disease-specific rather than universal. While NHP models remain invaluable in influenza research, particularly for investigating systemic immune activation, acute lung injury, and vaccine immunogenicity, their capacity to replicate IAE-related pathology appears constrained.

**Table 2:** Summary of (brain) pathological changes induced by IAV infection in different study models.

Research Models	Modes of Infection	Cell/Animal Types	Virus Strains	Pathological Characteristics	References
Cell lines	IAV directly infects neurons	Human SH-SY5Y	A/Hong Kong /457421/2009 (H1N1), A/Hong Kong/1750/2011 (H1N1), A/Hong Kong/54/98 (H1N1), A/Hong Kong/483/97 (H5N1), A/Vietnam/3212/04 (H5N1)	Capable of infecting cells and inducing an inflammatory response, but unable to produce infectious progeny viruses	[68,110]
			A/Shanghai/2/2013 (H7N9)	Capable of infecting cells, inducing high expression of pro-inflammatory cytokines and generating new virion progeny	[68]
		Human SK-N-SH	A/Indonesia/5/2005 (H5N1), A/Netherlands/213/2003 (H3N2), A/WSN/33 (H1N1), A/Netherlands/602/2009 (H1N1)	Capable of infecting cells, and generating new virion progeny	[138]
		Primary mouse cortex neurons	A/Netherlands/602/2009 (H1N1), A/PR/8/34 (H1N1), A/Shantou/169/2006 (H1N1), A/Netherlands/213/2003 (H3N2)	Capable of infecting cells but with limited replication	[111,138]
			A/Indonesia/5/2005 (H5N1), A/WSN/33 (H1N1)	Capable of infecting and replicating efficiently within cells	[138]
	IAV directly infects astrocytes	Human T98G	A/Hong Kong /457421/2009 (H1N1), A/Hong Kong/1750/2011 (H1N1), A/Shanghai/2/2013 (H7N9)	Capable of inducing cellular infection and promoting the release of inflammatory mediators but unable to produce new virion progeny	[108]
			A/Hong Kong/54/98 (H1N1), A/Hong Kong/483/97 (H5N1), A/Vietnam/3212/04 (H5N1)	Capable of infecting cells, and generating new virion progeny	[108,110]
		Human U87-MG	A/Netherlands/602/2009 (H1N1), A/Netherlands/213/2003 (H3N2)	Capable of infecting cells but with limited replication	[138]
			A/Indonesia/5/2005 (H5N1), A/WSN/33 (H1N1), A/Hong Kong/54/98 (H1N1), A/Hong Kong/483/97 (H5N1), A/Vietnam/3212/04 (H5N1)	Capable of infecting cells, and generating new virion progeny	[110,138]
		Primary mouse astrocytes	A/Shantou/69/06 (HN1N), A/Chicken/Guangdong/1/05 (H5N1)	Capable of inducing cellular infection, highly expressing inflammatory cytokines and replicating efficiently within cells	[139]
	IAV directly infects microglias	Mouse BV-2	pdm09 (H1N1), A/Shenzhen/13/2013 (H7N9)	Capable of infecting cells and producing infectious progeny	[139,140]
		Primary mouse microglias	A/Shantou/69/06 (HN1N), A/Chicken/Guangdong/1/05 (H5N1)		
Brain organoids	IAV directly infects human brain organoids	Human brain organoids	A/WSN/33 (H1N1)	Neurotoxic effects and apoptosis on infected NSCs and neurons, inducing brain organoids damage	[113]
Mouse models	Intranasal inoculation	C57BL/6J, BALB/c	A/PR/8/34 (H1N1)	Viral infection can induce cognitive and memory function impairment and affect long-term behavioral capabilities	[114,119,141]
		C57BL/6J	rSC35M, mouse-adapted A/Seal/Mass/1/80 (H7N7)	Long-term neurological damage, morphological alterations in hippocampal neurons, cognitive deficits	[120]
			maHK68, mouse-adapted A/ Hong Kong/1/68 (H3N2)		
		BALB/c	A/Chicken/Guangdong/V/2008 (H9N2)	Peripheral infections induce inflammation in brain	[116]
			A/Guangdong/GZ8H002/2017 (H7N9)	The virus can invade brain tissue in 7 dpi and cause severe infection	[115]
	Intravenous infection	C57BL/6	A/PR/8/34 (H1N1)	Infecting brain endothelial cells, inducing necrotic apoptosis, and cerebral edema	[67]
	Intraperitoneal inoculation	BALB/c	A/WSN/33 (H1N1), A/NWS/33 (H1N1)	Viral antigens were detected in the brain tissue of 5-day-old newborn mice and affected myelin formation	[73,79]
Ferret models	Intranasal inoculation	Ferrets	A/Hong Kong/483/1997 (H5N1)	No viral antigens were detected in brain tissue, but the infection caused brain tissue damage, such as vasculitis and haemorrhage	[81]
			A/Netherlands/177/2008 (H3N2)	No viral antigens were detected in brain tissue	[127]
			A/Hong Kong/486/1997 (H5N1)	Detectable viral antigens in brain tissue, with severe brain damage including choroiditis and vasculitis	[81]
			A/Netherlands/602/2009 (pH1N1), A/Indonesia/5/2005 (H5N1), A/Vietnam/1203/2004 (H5N1), A/Vietnam/UT3062/2004 (H5N1)	Detectable viral antigens in brain tissue	[127,132]
	Intragastric inoculation		A/Vietnam/1203/2004 (H5N1),	No viral antigens were detected in brain tissue	[132]
			A/Vietnam/UT3062/2004 (H5N1)	Detectable viral antigens in brain tissue	
Hamster models	Intranasal inoculation	Syrian hamsters	A/California/04/2009 (H1N1)	No viral antigens were detected in brain tissue	[133]
		Golden Syrian hamsters	A/Vietnam/1203/2004 (H5N1)		[132]
	Intragastric inoculation		A/Vietnam/1203/2004 (H5N1), A/Vietnam/UT3062/2004 (H5N1)		
Non-human primate models	Intranasal inoculation	Rhesus monkeys	A/whooper swan/Hokkaido/1/2008 (H5N1 clade 2.3.2.1)	Difficulty in detecting viral antigens in the brains	[135,136]
		Cynomolgus macaques	A/California/04/09 (H1N1)		
		Cynomolgus monkeys	A/whooper swan/Hokkaido/1/2008 (H5N1 clade 2.3.2.1)	No viral antigens were detected in brain tissue, only presence in the cerebellum	[135]

## 5. Summary

Influenza viruses primarily cause acute respiratory infections, resulting in significant morbidity and mortality worldwide. Influenza-associated encephalopathy/encephalitis (IAE) constitutes a major cause of influenza-related fatalities. However, definitive diagnosis of IAE is typically made only after pathological changes have occurred in the brain. The absence of early rapid diagnostic tools hinders the timely identification of critical pathological conditions, leading to delayed treatment and adverse outcomes. In recent years, increasing clinical case analyses and basic research have revealed that elevated levels of inflammatory cytokines within the body and direct influenza virus infection of the CNS are key mechanisms in pathogenesis of IAE. Furthermore, cytokine- and pathogen-induced glial cell activation, alongside pre-existing genetic susceptibility factors in individuals, also contribute to varying degrees to the development and progression of IAE to varying degrees.

## Figures and Tables

**Table 1:** Diagnosis, clinical manifestations, and prognosis of clinical syndromes of IAE.

Clinical Subtypes of IAE	Clinical Characteristics	Disease Outcomes	References
ANE	MRI: Multiple, symmetrical brain lesions involving thalamus, brainstem, cerebellum, and periventricular white matter	Rapid onset and deterioration, poor prognosis	[22,23,24,25,26,27]
AESD	MRI: Reduced subcortical diffusion and appearance of bright tree-like appearance	No preexisting brain abnormalities, but rapid deterioration and lack of early diagnostic indicators	[28,29,30,31,32,33]
MERS	MRI: Corpus callosum with splenial lesions	Recoverable brain lesions with good prognosis and no neurological sequelae	[34,35,36,37,38]
HSES	Diagnostic criteria of Bacon et al.: Symptoms of encephalopathy such as impaired consciousness and convulsions; shock; DIC; diarrhea; decreased hemoglobin concentration and platelet count; acidosis; elevated hepatocellular enzymes; renal impairment; negative blood and cerebrospinal fluid cultures	Rapid progression, poor prognosis, prone to sequelae, high mortality rate of 35–82%	[39,40,41,42]
PERS	MRI: Bilaterally symmetrical vasogenic cerebral edema of the cortex and deep subcortical white matter, involving the occipital and parietal lobes	Overall prognosis is good, clinical symptoms and imaging lesions are reversible	[43,44,45,46]
ADEM	Brain MRI: Multiple lesions in subcortical white matter or deep but not periventricular white matter Spinal cord MRI: Lesions in the spinal cord vertebral segments	The long-term prognosis is good, with clinical symptoms and MRI lesions generally improving within 3 months	[47,48,49,50,51]

## Data Availability

No new data were created or analyzed in this study.

## Abbreviations

The following abbreviations are used in this manuscript:

IAE	Influenza-associated encephalitis/encephalopathy
MRI	Magnetic resonance imaging
IAV	Influenza A virus
IBV	Influenza B virus
ICV	Influenza C virus
IDV	Influenza D virus
IVIG	intravenous immunoglobulin
EEGs	Electroencephalograms
ANE	Acute necrotizing encephalopathy
AESD	Acute encephalopathy with biphasic seizures and late diffusion restriction
MERS	Mild encephalitis/encephalopathy of a reversible splenial lesion
HSES	Hemorrhagic shock and encephalopathy syndrome
PRES	Posterior reversible encephalopathy syndrome
ADEM	Acute disseminated encephalomyelitis
DIC	Disseminated intravascular coagulation
MOG	Myelin-associated glycoprotein
NMO	Neuromyelitis optical
TNF	Tumor necrosis factor
IFN	Interferon
IL	Interleukin
BBB	Blood–brain barrier
CNS	Central nervous system
ZO-1	Zonula occludens-1
MHC	Major histocompatibility complex
C3	Complement component 3
CSF	Cerebrospinal fluid
HPAIV	Highly pathogenic avian influenza viruses
MBCS	Multibasic cleavage site
*CPT II*	Carnitine palmitoyltransferase II
*ADORA2A*	Adenosine A2A receptor gene
SNPs	Single-nucleotide polymorphisms
*STK39*	Serine/threonine kinase 39 gene
MAPK	Mitogen-activated protein kinase
*RANBP2*	RAN binding protein 2
hPSCs	Human pluripotent stem cells
NSCs	Neural stem cells
IBA-1	Ionized calcium-binding adapter molecule 1
BDNF	Brain-derived neurotrophic factor
PrP^C^	Cellular prion protein
PrP^SC^	Scrapie prion protein
VN1203	A/Vietnam/1203/2004 (H5N1)
UT3062	A/Vietnam/UT3062/2004 (H5N1)
SARS-CoV-2	Severe acute respiratory syndrome coronavirus 2
NHP	Non-human primates
